# Identification and validation of three tumor suppressors associated with the immune response of acute myeloid leukemia

**DOI:** 10.3389/fgene.2025.1652142

**Published:** 2025-09-16

**Authors:** Yueyuan Pan, Guocai Wu, Chenchen Liu, Minggui Chen, Tian Xia, Yonghua Ma, Zhigang Yang, Ruiting Wen

**Affiliations:** ^1^ Zhanjiang Institute of Clinical Medicine, Central People’s Hospital of Zhanjiang, Zhanjiang, China; ^2^ Department of Hematology, Central People’s Hospital of Zhanjiang, Zhanjiang, China; ^3^ Zhanjiang Key Laboratory of Leukemia Pathogenesis and Targeted Therapy Research, Zhanjiang, China; ^4^ Precision clinical laboratory, Central People’s Hospital of Zhanjiang, Zhanjiang, China

**Keywords:** acute myeloid leukemia, tumor immune microenvironment, Weighted Gene Co-expression Network Analysis, tumor suppressor, immune response

## Abstract

**Background:**

Acute myeloid leukemia (AML) is a heterogeneous disorder marked by irregular expansion and maturation, giving rise to the aggregation of immature myeloid precursor cells. Although most patients achieve remission with initial treatment, the majority of relapses lead to poorer overall survival. The bone marrow (BM) immune microenvironment has been proven to significantly affect the progression of AML. However, the mechanisms that cause the imbalance of immune cell subsets and phenotypes remain partially obscure. Therefore, this research sought to explore the immune-regulatory genes and to determine their role in AML.

**Methods:**

Differentially expressed genes (DEGs) were obtained through differential analysis of the AML cohort. Enrichment analyses were applied to explore their biological functions. Weighted Gene Co-expression Network Analysis (WGCNA) was performed to identify the key module of AML. ROC curve analysis was performed to identify hub genes with good predictive power. CIBERSORT and the ESTIMATE algorithm were used to assess the correlation between hub genes and the immune microenvironment of AML. The impact of hub gene expression on the prognosis of AML was verified through prognostic traits and clinical samples.

**Results:**

Through differential analysis and WGCNA, seven genes were identified as markedly related to the development of AML. By mapping ROC curves, three hub genes were verified: CCR7, SLC16A6, and MS4A1, which have high diagnostic value for AML. Additionally, an imbalanced immune microenvironment was found to be common in AML. Three hub genes were significantly associated with immune components, including immune cells and immunomodulatory factors. Ultimately, through the validation of clinical samples and the analysis of prognostic characteristics, three genes were confirmed to be reduced in AML patients, and their high expression suggested a favorable prognosis.

**Conclusion:**

Our study identified and validated the efficacy of SLC16A6, CCR7, and MS4A1 as tumor suppressors implicated in AML progression and related to immune cell infiltration.

## 1 Introduction

Acute myeloid leukemia (AML) is marked by the accumulation of naive cells, caused by abnormal differentiation and proliferation of the myeloid lineage (Forsberg and Konopleva, 2024). Although the complete remission rate is 40%–80% for patients, the overall survival rate remains low (Kantarjian, 2016; McNerney et al., 2017; Dohner et al., 2010; Dombret and Gardin, 2016). Recent research indicates that AML is intimately linked to the tumor immune microenvironment (TIME) (Subklewe et al., 2023; Lamble and Lind, 2018; Vadakekolathu and Rutella, 2024). AML can shape the TIME by interacting with immune cells, leading to alterations in their activity and phenotype (Perzolli et al., 2024). It has been demonstrated to induce suppressive populations, like myeloid-derived suppressor cells (MDSCs); regulatory T cells (Tregs), which dampen the function of cytotoxic T cells; and natural killer (NK) cells (Park et al., 2022; Pyzer et al., 2017). Macrophages are the essential cellular component of the immunosuppressive TIME. Through the secretion of immunosuppressive enzymes or the activation of transcription factors, AML can directly induce macrophages to develop an M2-like phenotype, which suppresses T-cell proliferation and function (House et al., 2020; Hoch et al., 2022). In the bone marrow (BM) of AML, M2-like macrophages negatively correlate with T-cell infiltration, with poor prognosis (Koedijk et al., 2024). In addition, exhausted T cells accumulate in the tumor microenvironment (TME) and manifest defective killing capacity (Voehringer et al., 2002).

The modulation of TME is also a great challenge for the successful translation of novel immunotherapies. An effective strategy involves focusing on the BM niche to counteract the immunosuppressive microenvironment, which includes two primary methods: diminishing the quantity of immunosuppressive cells and repolarizing them toward an anti-tumor phenotype (Vatner and Formenti, 2015). Meanwhile, reconstructing the cytotoxicity of effector cells (NK cells and T cells) is an effective immunotherapy for AML (9). Although the molecular genetics of AML have been thoroughly examined, the interaction between the immune response and genetic alterations remains incompletely understood. It is essential to investigate biomarkers related to immune infiltration in AML to clarify their connection with the tumor microenvironment and disease traits. In this research, we identified three immune-regulatory genes linked to the emergence and progression of AML and explored their association with the immune milieu.

## 2 Methods

### 2.1 AML dataset acquisition and processing

We retrieved the AML datasets [GSE9476 (Stirewalt et al., 2008) and GSE114868 (Huang et al., 2019)] from the Gene Expression Omnibus (GEO) database. Samples were grouped based on the information provided by authors. GSE9476 comprises 20 normal controls and 26 AML patient samples. GSE114868 comprises 20 normal controls and 194 AML patient samples. Both cohorts have been normalized.

### 2.2 Differential analysis and functional enrichment

R version 4.3.3 was used to complete this research. Differentially expressed genes (DEGs) of AML were obtained using the package “Limma” (McCarthy and Smyth, 2009). The package “ClusterProfiler” was applied to perform enrichment analysis (Yu et al., 2012). Finally, the package “ggplot2” was used to generate images for visualization.

### 2.3 Immune cell infiltration and immunity index score analysis

The package “ESTIMATE” was used to assess stromal scores and immune scores (Yoshihara et al., 2013). “CIBERSORT” was applied to examine the extent of immune cell infiltration within each sample (Newman et al., 2015), based on the known leukocyte expression matrix LM22, and the permutations (PERM) were set to 1,000 to obtain reliable results.

### 2.4 Correlation analysis

Correlation analysis was performed using the “Corrplot” package.

### 2.5 WGCNA

Weighted Gene Co-expression Network Analysis (WGCNA) was applied to explore the crucial module of AML (Langfelder and Horvath, 2008). We used the genes with coefficients of variation in the top 25% as input data. A soft-threshold value of 14 was selected to ensure the construction of a stable and reliable co-expression network. The dynamic tree-cutting algorithm was used to aggregate genes with similar biological characteristics into the same module. Based on the association index between the module and AML, the key modules of AML were identified.

### 2.6 Identification of hub genes

Seven overlapping genes were obtained by intersecting the three candidate gene sets. The “pROC” package was applied to construct the ROC curve (Robin et al., 2011). The candidate genes were ranked by their AUC values, with the top three designated as hub genes.

### 2.7 Prognostic analysis of three genes

Using the threshold determined by the minimum p-value from the log-rank test, AML patients were split into groups with high and low expression levels. The Kaplan–Meier survival curve was then generated to evaluate survival differences between these two groups (Gyorffy, 2024). The GSE76008 cohort was used to clarify the differences in hub genes between leukemia stem cell (LSC)-positive and LSC-negative cells (Ng et al., 2016). The GSE83533 cohort was used to investigate the differences between diagnostic and relapsed AML samples (Li et al., 2016).

### 2.8 Isolation of bone marrow mononuclear cells (BMNCs)

The acquisition and utilization of the samples were performed in accordance with the principles of the Declaration of Helsinki. Bone marrow was collected from AML patients and healthy donors of allogeneic hematopoietic stem cell transplantation patients at the Central People’s Hospital of Zhanjiang, and we used human lymphocyte separation medium to isolate the bone marrow mononuclear cells (BMNCs) according to the manual. BMNCs were placed in TRIzol for preservation.

### 2.9 Real‐time quantitative polymerase chain reaction (qRT-PCR)

Real‐time quantitative polymerase chain reaction (qRT-PCR) was applied to evaluate the difference in hub genes between healthy controls and AML patients. Total RNA was isolated using TRIzol reagent, and 1 μg of RNA was reverse-transcribed into stable complementary DNA (CDNA) using reverse transcriptase. A volume of 20 µL of qPCR reaction mixture was prepared, containing SYBR, primers, nuclease-free water, and template cDNA. After obtaining the CT values, relative gene expression was assessed using the comparative CT method [2^(-ΔΔCT)^]. The primer sequences used were as follows:

**Table:** 

Gene	Forward	Reverse
GAPDH	ACA​ACT​TTG​GTA​TCG​TGG​AAG​G	GCC​ATC​ACG​CCA​CAG​TTT​C
SLC16A6	CGC​TGT​GTT​TGC​TTT​CGC​ACC​A	TTT​TCG​GTG​ACG​CTG​GTC​CTC​T
CCR7	CAA​CAT​CAC​CAG​TAG​CAC​CTG​TG	TGC​GGA​ACT​TGA​CGC​CGA​TGA​A
MS4A1	CTG​GTC​CAA​AAC​CAC​TCT​TCA​GG	GGC​AAT​GTG​GAA​GAG​CCC​ATT​C

### 2.10 Statistical analysis

Statistical analyses in R were performed using the “ggpubr” package, and images were generated using “ggplot2.” SPSS 19.0 was used for statistical analysis. An independent-samples t-test or a Mann–Whitney U test was selected based on whether the sample conformed to a normal distribution and whether variances between groups were equal. All data were presented as mean ± standard error of the mean (SEM). The images were edited using Adobe Photoshop (PS) 2022 software.

## 3 Results

### 3.1 Flowchart of this study

**FIGURE 1:**
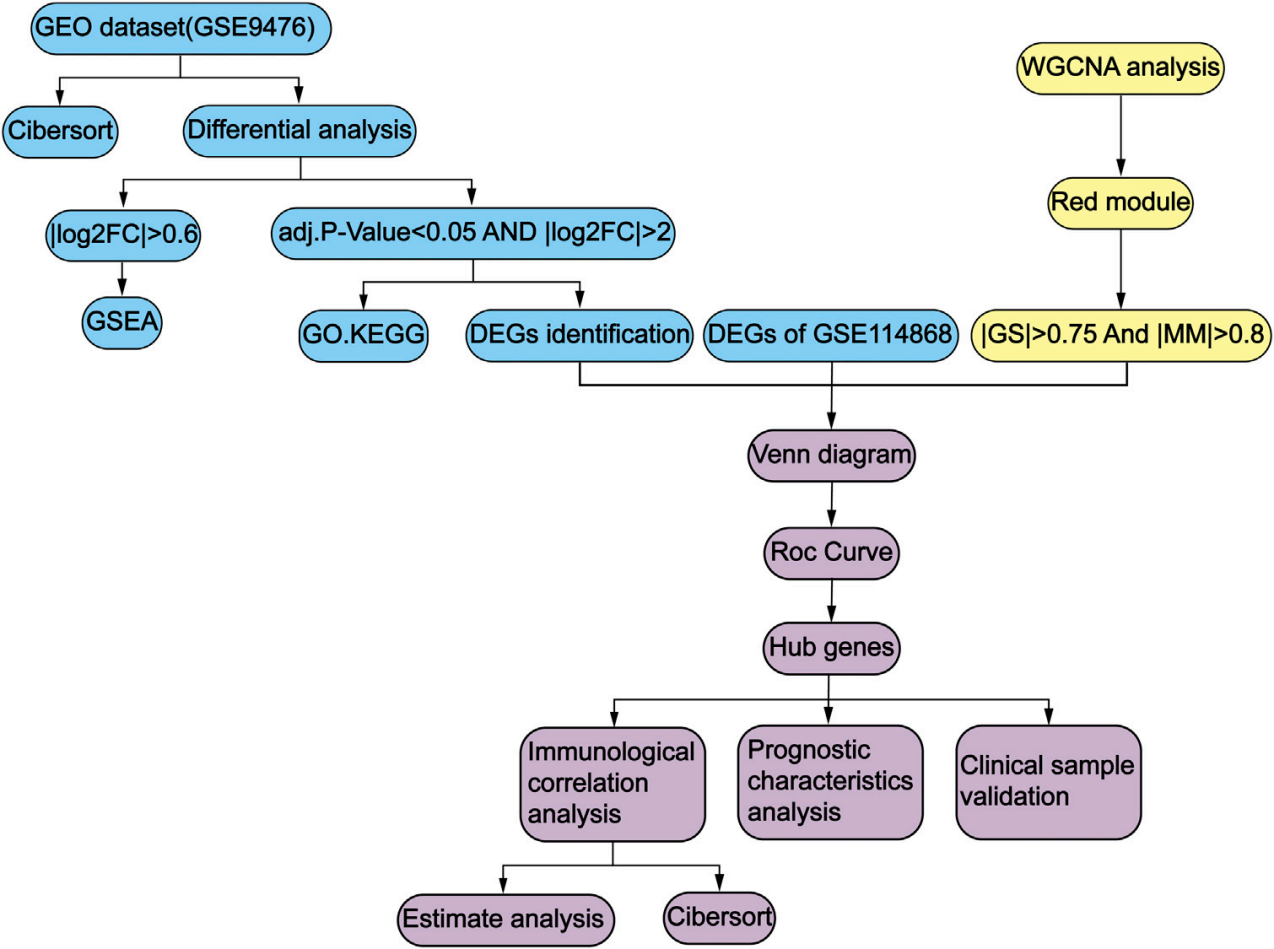
Flowchart for this study.

Figure 1 illustrates the overall workflow of this study. GSE9476 and GSE114868 datasets were used to investigate biomarkers of AML. Through differential analysis, WGCNA, and ROC curve analysis, we identified three immune-related hub genes. Furthermore, through prognostic analysis, immune-infiltration profiling, and validation in clinical samples, we clarified the critical roles of these hub genes in the initiation and progression of AML.

### 3.2 Immune response involved in the development of AML

**FIGURE 2:**
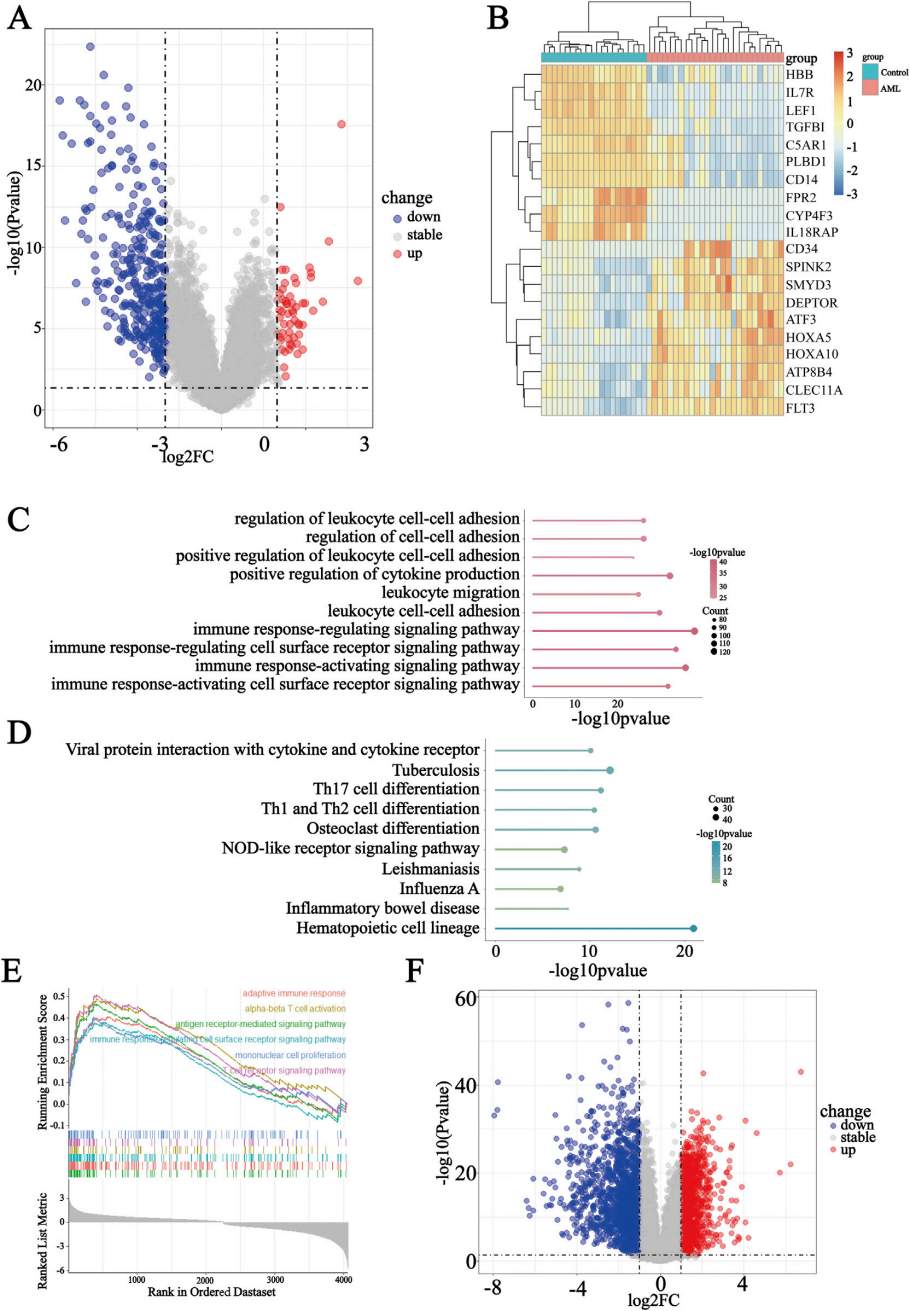
Identification of AML-specific expression profiles. **(A)** Volcano plot displaying the number and spread of all DEGs in GSE9476. **(B)** Heatmap depicting the expression of the top 20 DEGs across the two groups. **(C–E)** Enrichment analyses suggesting a variety of biological processes in which DEGs are involved, including GO terms, KEGG, and GSEA. **(F)** Volcano plot displaying the number and spread of all DEGs in GSE114868.

The GSE9476 dataset, comprising 20 normal controls and 26 AML samples, was utilized to explore DEGs between the two groups. In total, 1,389 DEGs were obtained, characterized by |log2FC|>1 and adj. p-value<0.05, comprising 450 upregulated genes and 939 downregulated genes (Figure 2A). A heatmap was used to demonstrate the top 10 upregulated genes, namely, CD34, SPINK2, SMYD3, DEPTOR, ATF3, HOXA5, HOXA10, ATP8B4, CLEC11A, and FLT3, whereas the expressions of IL18RAP, CYP4F3, FPR2, CD14, PLBD1, C5AR1, TGFB1, LEF1, IL7R, and HBB were downregulated in AML (Figure 2B). Gene Ontology (GO) Enrichment Analysis clarified that these DEGs were prominently involved in immune response pathways (Figure 2C). KEGG analysis confirmed that hematopoietic cell lineage and Th1 and Th2 cell differentiation were highly associated with AML (Figure 2D). Moreover, Gene Set Enrichment Analysis (GSEA) also suggested that the immune processes may be markedly involved in AML (Figure 2E). Genes with the criteria of |log2FC|>2 and adj. p-value <0.05 in GSE9476 were considered candidate gene set A. Furthermore, to identify feature genes pivotal for the initiation and progression of AML, the GSE114868 dataset was additionally incorporated into the analysis. Differential expression analysis of this dataset yielded a total of 2,850 DEGs (|log2FC|>1 and adj. p-value<0.05), comprising 1,304 upregulated genes and 1,546 downregulated genes (Figure 2F). Finally, genes with the criteria of |log2FC|>3.5 and adj. p-value <0.05 of GSE114868 were considered candidate gene set B.

### 3.3 Imbalance between immunosuppressive cells and pro-inflammatory cells in AML

**FIGURE 3:**
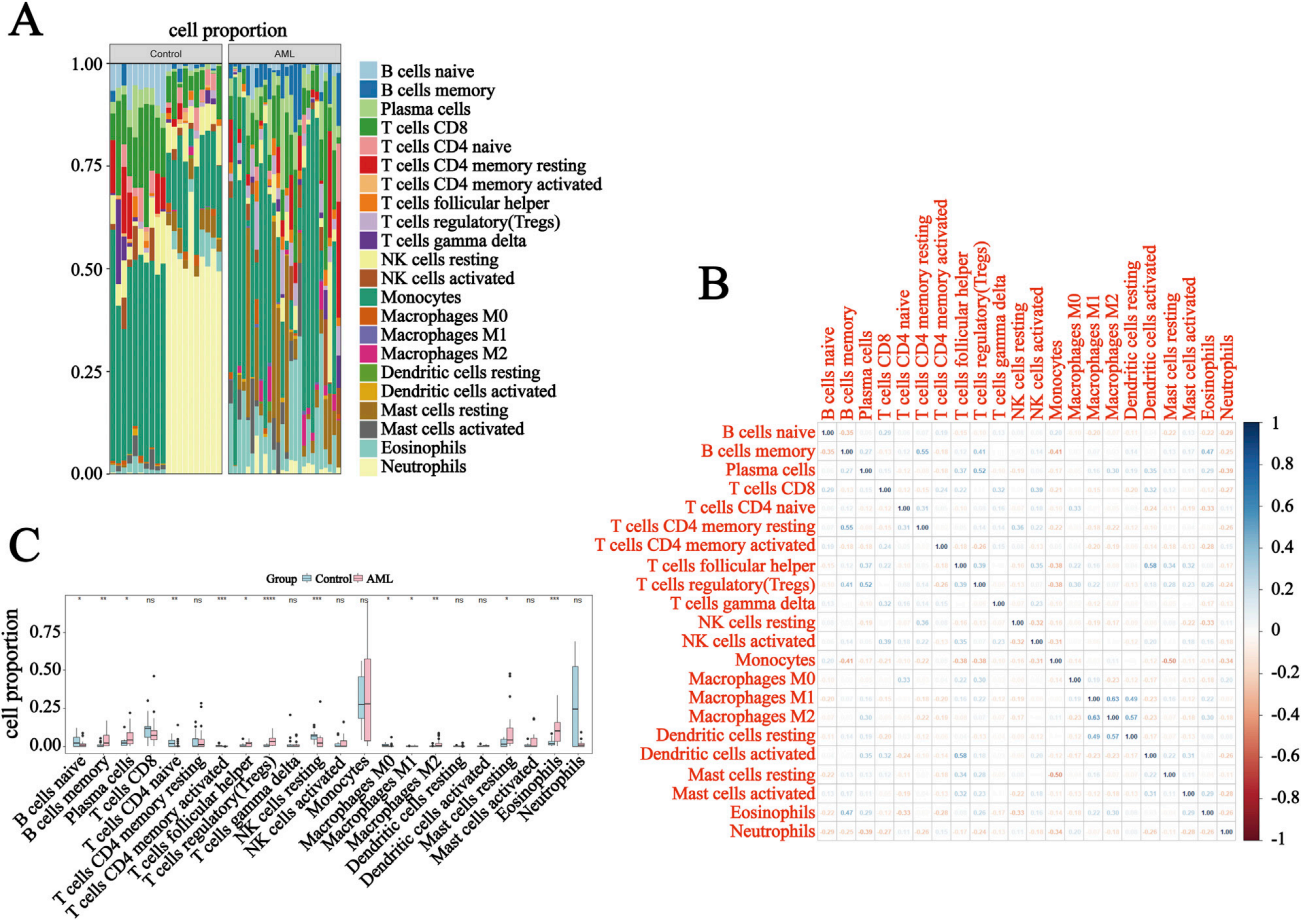
The immune landscape of the training dataset GSE9476. **(A)** Stacked bar plot presenting the percentage distribution of 22 immune cell types in each sample. **(B)** Heatmap illustrating the correlation among each immune cell. **(C)** The expression levels of the 22 immune cell types in normal controls and AML in GSE9476. *P < 0.05, **P < 0.01, and ***P < 0.001.

Compared with the normal controls, AML was characterized by the enrichment of immune-related pathways. Therefore, the CIBERSORT algorithm was applied to explore the immune landscape in the training dataset GSE9476. A stacked bar plot and a heatmap displayed the proportions of immune cells and correlations among 22 immune cell types (Figures 3A,B). We next analyzed the immune cell infiltration in each sample. It was suggested that AML exhibited increased infiltration by immunosuppressive cells (Tregs, M2-like macrophages, plasma cells, and resting mast cells), while the fraction of pro-inflammatory cells (M1-like macrophages and activated CD4^+^ memory T cells) and naïve T/B cells were significantly reduced (Figure 3C), which were characterized by the immunosuppressive microenvironment. These results suggested an imbalance between immunosuppressive and pro-inflammatory cells in AML, which further shaped the dysfunctional immune microenvironment.

### 3.4 Identification of the key immune-related gene module of AML by WGCNA

**FIGURE 4:**
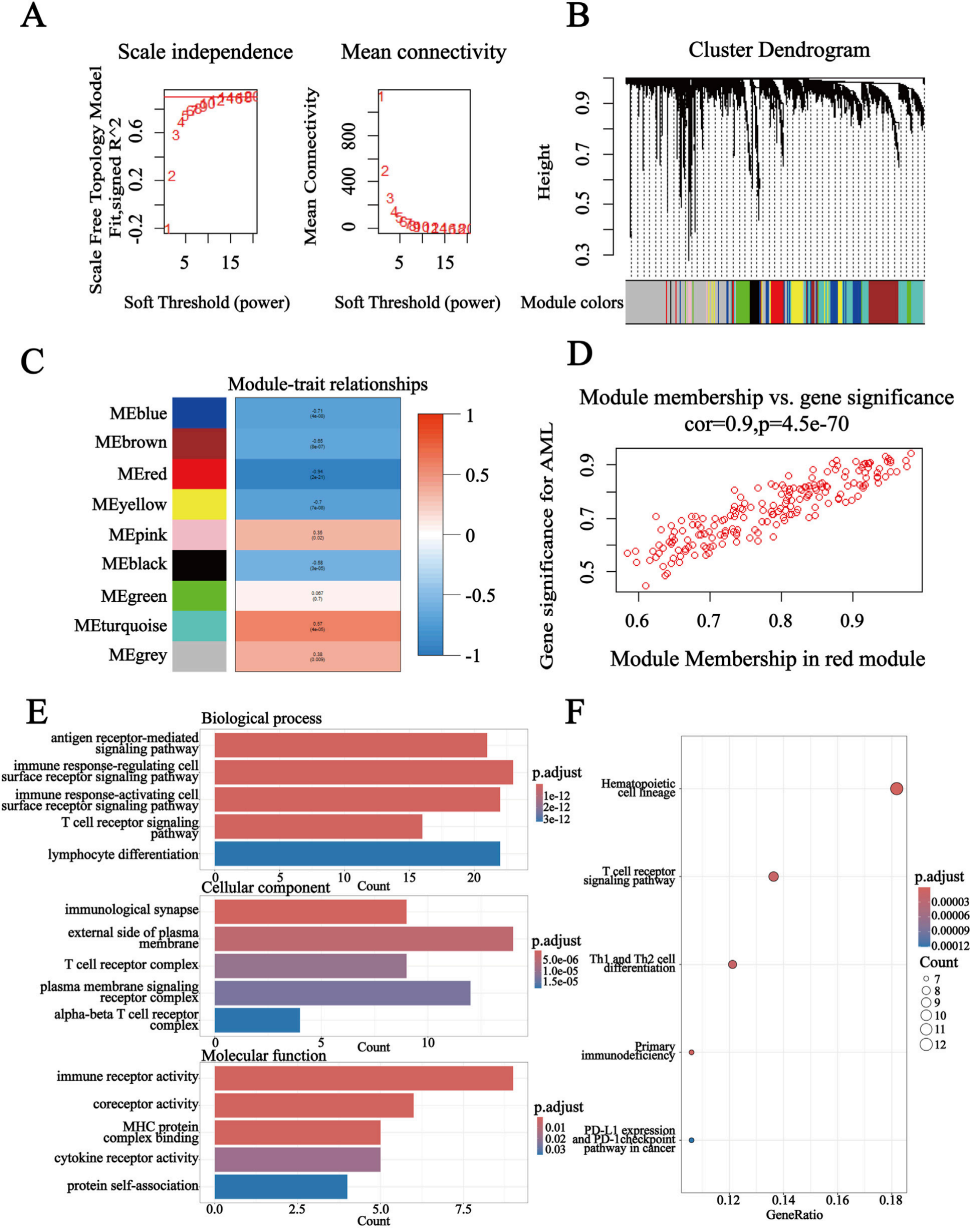
WGCNA of GSE9476. **(A)** Network topology analysis selected the optimal soft threshold to establish a co-expression network. **(B)** Applying the dynamic cutting method to construct hierarchical clustering trees, and genes exhibiting resembling expression models were clustered into a gene module. **(C)** Heatmap illustrating the correlation and P-value between gene modules and AML. **(D)** The correlation coefficient between AML and red modules was 0.9, revealing that the module was significantly related to AML. **(E)** GO analysis of the red module, including biological process (BP), cellular component (CC), and molecular function (MF). **(F)** KEGG analysis of the red module, further indicating that these genes participated in various processes.

WGCNA was applied to identify the key module of AML in GSE9476. A robust co-expression network was established using the power of 14 (Figure 4A). Based on hierarchical clustering and the principle of dynamic tree cutting, a clustering dendrogram was constructed (Figure 4B). Genes with resembling expression models were clustered into a gene module, resulting in a total of nine gene modules, and the red module was the most significant (*R* ^2^ = 0.94, P < 0.001) related to AML (Figures 4C,D). We performed enrichment analyses on the genes in the red module to explore their potential biological functions. These genes were also notably abundant in immune pathways. The GO enrichment results mainly involved lymphocyte differentiation and immune receptor activity (Figure 4E). KEGG pathway analysis indicated that the DEGs potentially participated in hematopoietic cell lineage and T-cell receptor signaling (Figure 4F). Finally, genes with |GS|>0.75 and |MM|>0.8 in the red module were verified as candidate gene set C.

### 3.5 Identification of three hub immune-related genes in AML

**FIGURE 5:**
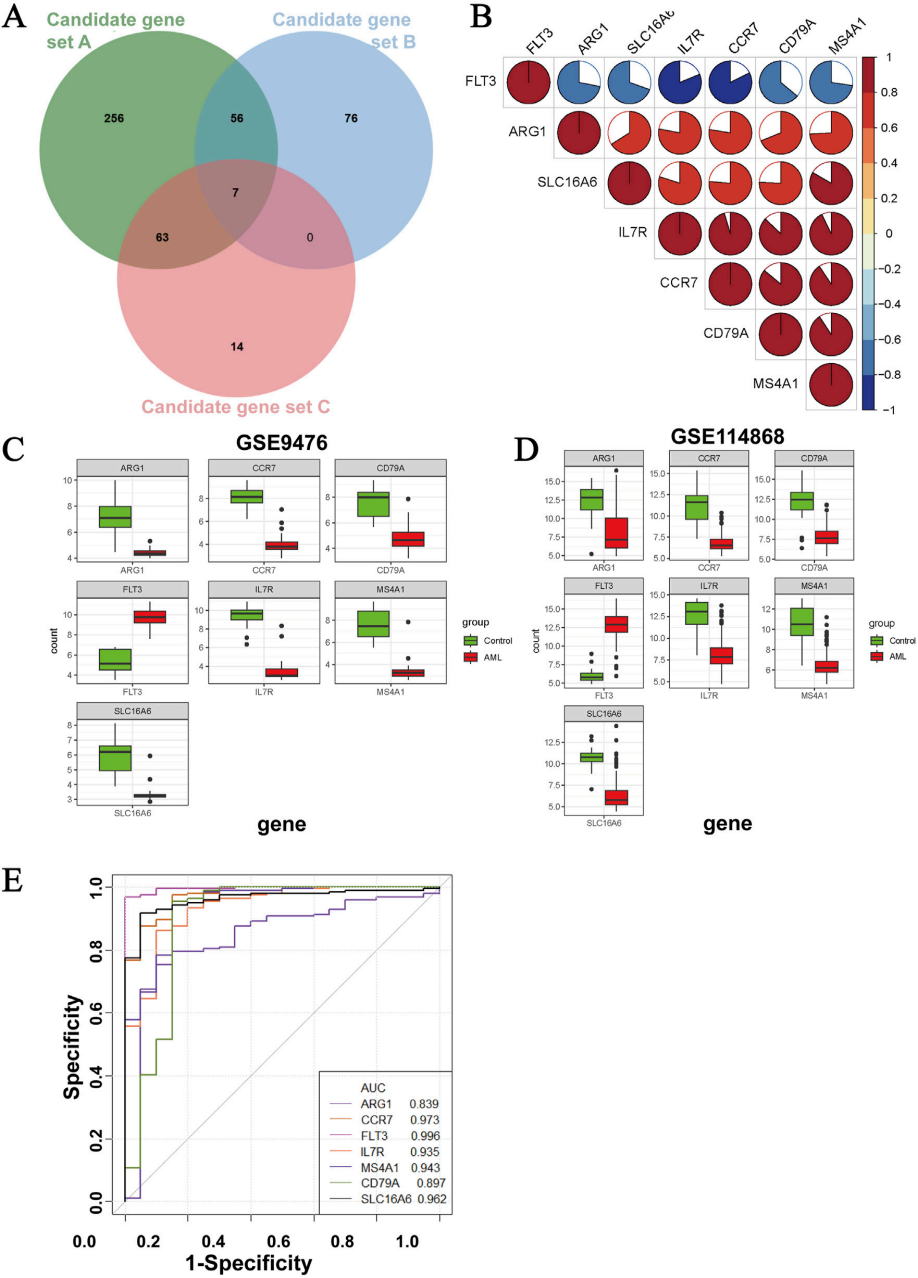
Screening for hub genes. **(A)** Venn diagram for three candidate gene sets. **(B)** Correlation analysis of candidate genes. **(C,D)** Expression levels of candidate genes in GSE9476 and GSE114868. **(E)** ROC curve analysis of candidate genes in GSE114868.

Seven key genes were obtained by intersecting the three candidate gene sets (Figure 5A), which were strongly correlated (Figure 5B). In the GSE9476 dataset, the expressions of CCR7, SLC16A6, MS4A1, CD79A, IL-7R, and ARG1 were markedly downregulated, while that of FLT3 was upregulated in AML samples (Figure 5C). The consistent findings were subsequently validated in the GSE114868 dataset (Figure 5D). The ROC curve can quantitatively evaluate the disease diagnostic ability of indicators; a higher AUC value indicates stronger diagnostic performance. In some studies, it had been applied to screen for biomarkers (Gong et al., 2025; Li et al., 2025). We applied the ROC curve to assess the diagnostic ability of the seven key genes (Figure 5E). Ranked by the AUC value, the highest AUC was FLT3, followed by CCR7, SLC16A6, MS4A1, IL7R, CD79A, and ARG1. Given that the relationship between FLT3 and AML was well established, we selected the top three genes CCR7, SLC16A6, and MS4A1 as the final hub genes for further study.

### 3.6 Substantial association between three genes and the immune microenvironment

**FIGURE 6:**
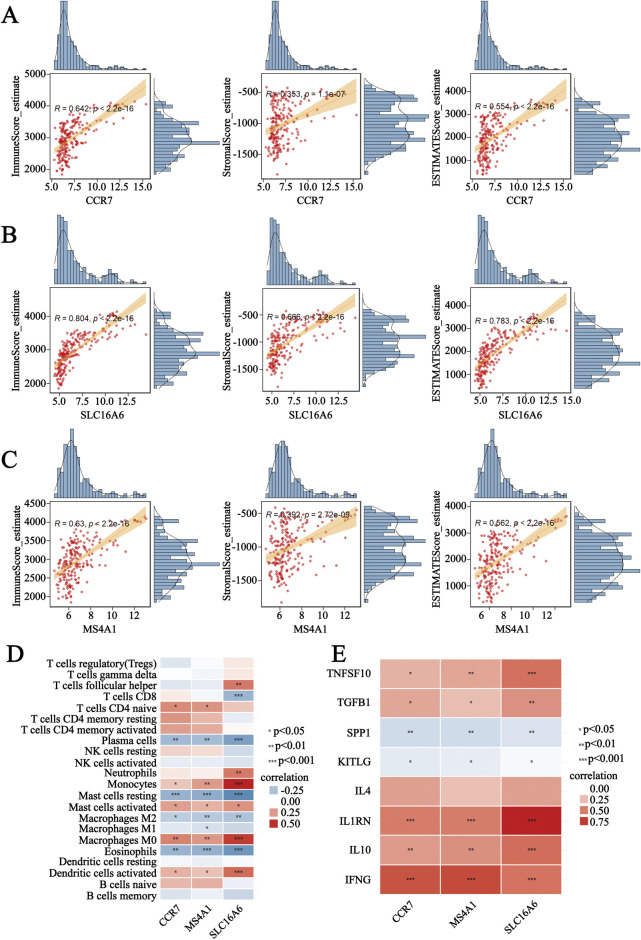
Analysis of the correlation between three genes and the immune microenvironment. **(A–C)** Correlation analysis between three genes and immune, stromal, and estimate scores. **(D)** Heatmap showing correlation between three genes and 22 types of immune cells. *P < 0.05, **P < 0.01, and ***P < 0.001. **(E)** Correlation analysis of three genes with anti-oncogenic and oncogenic factors. *P < 0.05, **P < 0.01, and ***P < 0.001.

AML was characterized by an imbalance in the immune microenvironment and abnormality of the immune process (Figures 2, 3). We further investigated the role of hub genes in AML immunity. The ESTIMATE algorithm was applied to assess the correlation between three genes and the TIME. The results confirmed that CCR7, SLC16A6, and MS4A1 showed a strong positive association with the immune, stromal, and estimate scores, suggesting that these factors could be crucial in remodeling the TIME (Figures 6A–C).

The immune score merely indicated the overall quantity of infiltrating immune cells, but not the actual immune state within the TIME. Therefore, the CIBERSORT algorithm was used to analyze the associations between three genes and 22 types of immune cells in GSE114868. Further analysis revealed strong correlations between key biomarkers (SLC16A6, CCR7, and MS4A1) and specific immune cells. They were positively correlated with some of the pro-inflammatory cell types, such as activated dendritic cells (DCs), monocytes, and activated mast cells (Figure 6D). On the contrary, hub genes were negatively correlated with anti-inflammatory cell types like M2-like macrophages, resting mast cells, and plasma cells (Figure 6D), while these cells were significantly increased in AML (Figure 3). These findings indicated that hub genes might exert an anti-tumor effect by shaping the pro-inflammatory phenotype. In AML, imbalances in the intricate interplay between pro- and anti-inflammatory cytokines can create a tumor-promoting microenvironment that impacts the proliferation and survival of leukemia cells (Binder et al., 2018). A variety of immune-regulatory factors were selected to explore their associations with hub genes, which have been reported to have a definite relationship with AML (Luciano et al., 2022). As a result, hub genes were positively associated with anti-oncogenic cytokines (TNFSF10, TGF-β, IL4, IL1RN, IL10, and IFN-γ) in AML (Figure 6E). SPP1 and KITLG, as oncogenic factors in AML, were negatively correlated with hub genes (Figure 6E). The findings demonstrated that three genes may influence TIME by modulating immune cell infiltration and contributing to the regulation of cytokines.

### 3.7 High expression of hub genes indicated favorable prognosis for AML

**FIGURE 7:**
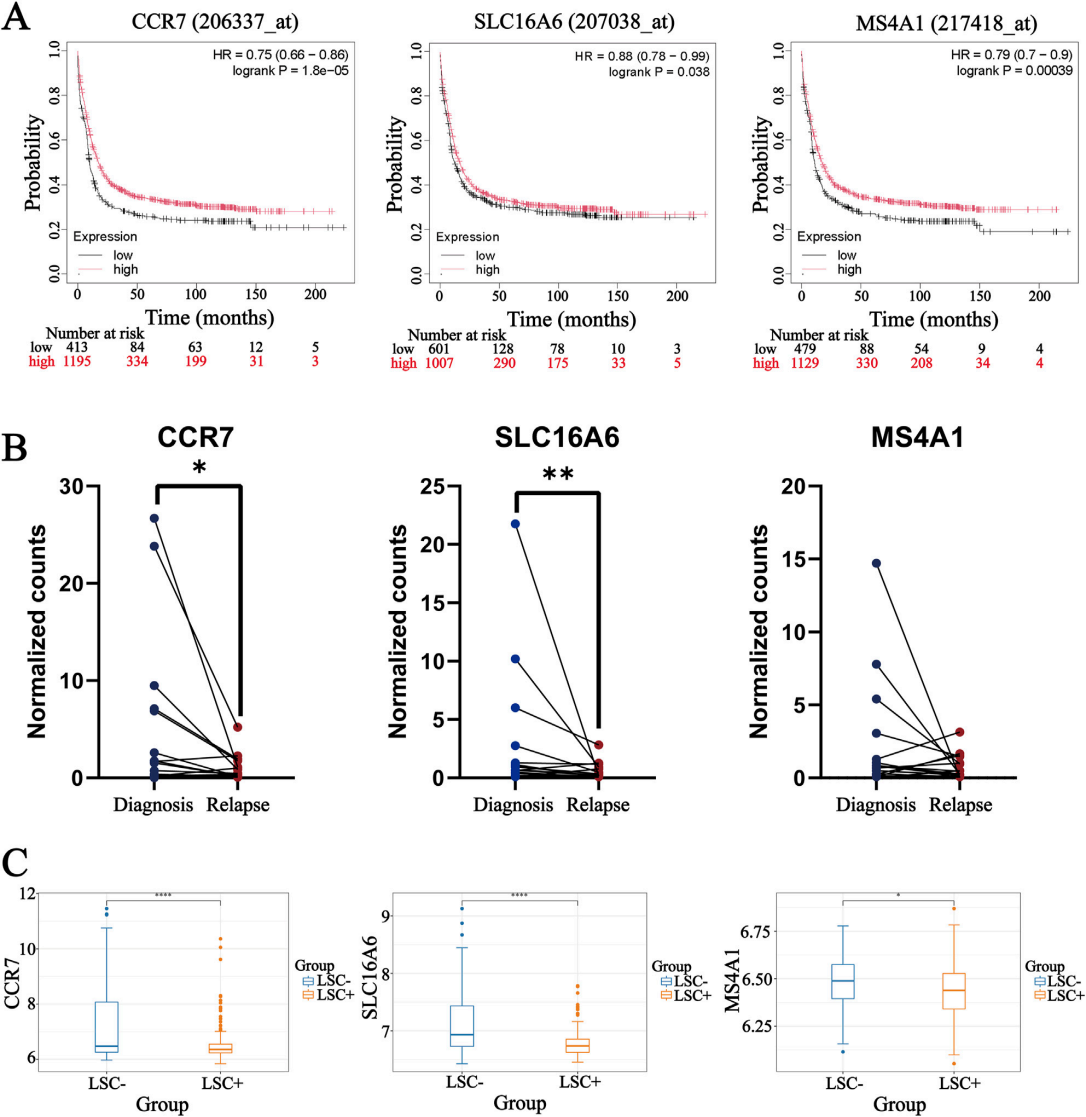
Exploration of the prognostic and clinical correlation of three genes in AML. **(A)** Kaplan–Meier survival curves assessing the prognostic value of three genes in AML. **(B)** Expression of three genes in diagnosed and relapsed patients. *P < 0.05 and **P < 0.01. **(C)** Differential expression of hub genes in LSC^+^ and LSC^−^ cells. *P < 0.05 and ****P < 0.0001.

As previously described, hub genes may affect the development of AML by reshaping the TIME. We next explored the association among three genes and prognosis from three aspects: survival curves, relevance to LSC, and recurrence. Patients with high expression of three genes consistently displayed better prognosis, whereas those with low expression typically faced shorter survival times (Figure 7A). Although most patients can achieve remission through initial treatment, most relapses lead to a poor overall survival. Therefore, recurrence is a key factor affecting the prognosis of AML. We compared the expression of three genes at the time of diagnosis and relapse from paired samples. The results confirmed that three genes were further decreased at relapse, and the trends of CCR7 and SLC16A6 were statistically significant (Figure 7B). Recurrence was usually driven by a rare subpopulation of LSC. Moreover, the result verified that three genes were significantly reduced in LSC^+^ cells (Figure 7C). These results suggested that hub genes may intervene in survival outcomes by regulating the activity of LSC subpopulations and affecting patient recurrence. Furthermore, we used the GSE37642 dataset to examine the associations between the three hub genes and the molecular characteristics of AML patients. As generally recognized, AML patients with RUNX1–RUNX1T1 fusion have a favorable prognosis (Sun et al., 2024); the expressions of CCR7 and MS4A1 were upregulated in this subgroup. Conversely, RUNX1 mutation denotes poor prognosis (Tang et al., 2009), and the expressions of both CCR7 and MS4A1 were downregulated in patients with RUNX1 mutations. Differential expression of the hub genes was also observed across distinct FAB subtypes of AML (Supplementary Figure 1).

### 3.8 Decreased expression of hub genes in AML clinical samples

**FIGURE 8:**
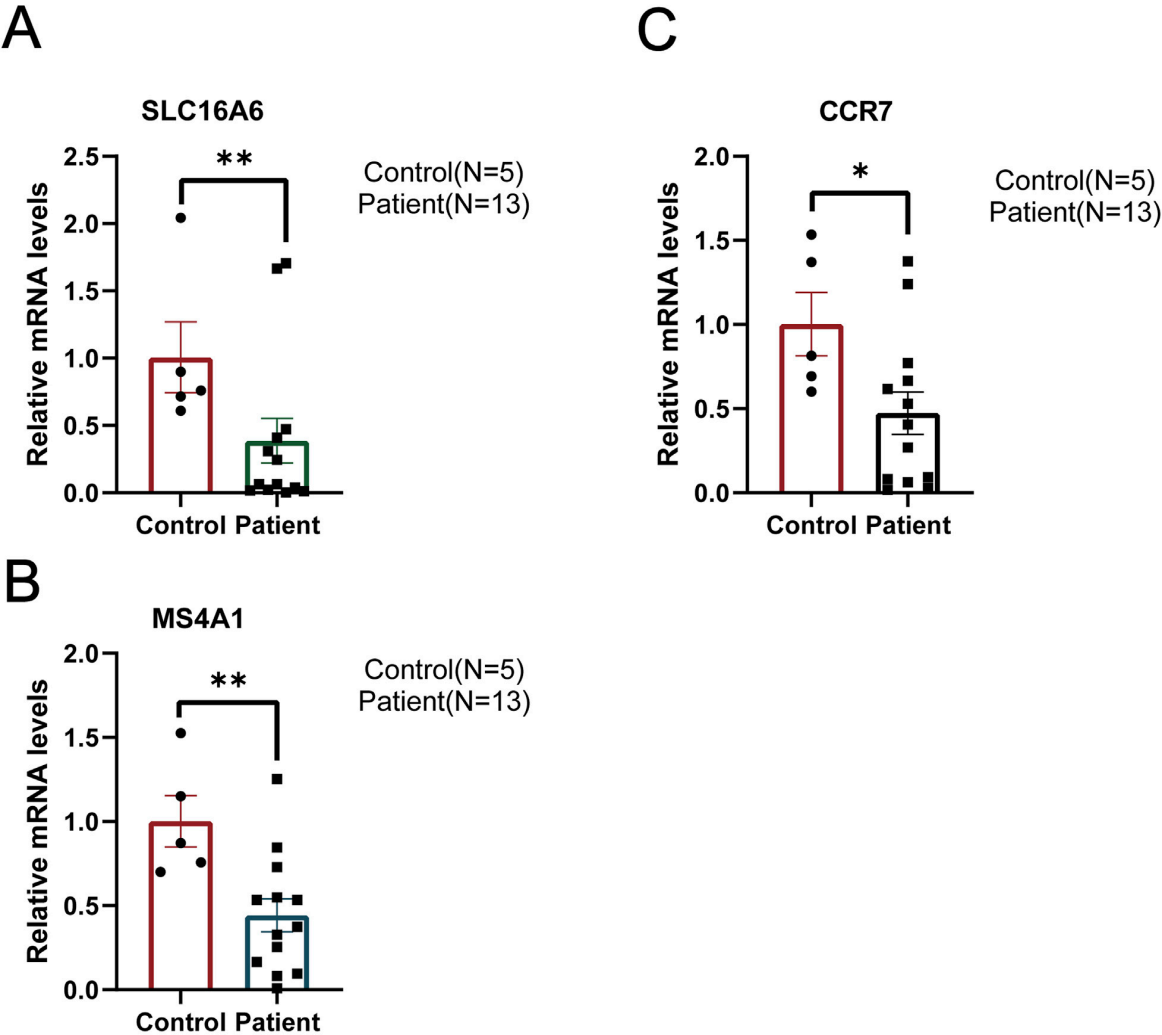
Detecting the expression of three genes in BMNCs of AML patients. **(A–C)** Relative mRNA expressions of SLC16A6, CCR7, and MS4A1 in BMNCs of healthy controls and AML patients were assessed using real-time fluorescence quantification. GAPDH served as the internal control for normalization. *P < 0.05 and **P < 0.01; mean ± SEM.

It is considered that the BM cell niche promotes leukemogenesis. We collected BMNCs from 5 healthy controls and 13 AML patients to detect the expression of three genes between two groups. The clinical and molecular characteristics of the 13 AML patients can be found in Supplementary Table 1. The results confirmed that, relative to healthy controls, these genes were markedly reduced in AML patients (Figures 8A–C). These results further indicated that three genes, as tumor-suppressor genes, were implicated in the onset and progression of AML.

## 4 Discussion

AML is a highly heterogeneous disease; although current therapies may achieve remission in some patients, the overall survival rate remains poor (Nair et al., 2021). The transformation of BM cells and the clonal growth of AML were highly related to the microenvironment, and immune microenvironment is crucial for the formation and progression of AML (Baryawno et al., 2019; Liu et al., 2022). However, the immune cell components and underlying mechanisms in the AML microenvironment remain incompletely understood. Therefore, to verify the immune-related biomarkers of AML is essential.

In our study, the enrichment analysis results from 26 AML patient samples and 20 healthy donor samples indicate that AML had characteristics of immune response dysregulation. We compared the differences in 22 types of immune cells between normal controls and AML patients and identified several distinct immune cells with varying expression levels between the two groups. Anti-inflammatory cells, like Tregs and M2-like macrophages, were upregulated in AML patients, while the expression of activated CD4^+^ T cells was significantly downregulated, leading to changes in the immune landscape, which aligns with prior research (Corradi et al., 2022; Weinhauser et al., 2023; Guo et al., 2021). These results suggested that there was an imbalance between immunosuppressive cells and pro-inflammatory cells in AML.

Through comprehensive bioinformatics analysis, we ultimately identified CCR7, SLC16A6, and MS4A1 as hub genes of AML, which had high diagnostic value and indicate prognostic traits related to AML. More importantly, hub genes were highly correlated with the immune microenvironment, mainly reflecting in their close association with various immune cells and immune-regulatory factors.

CCR7, as a chemokine receptor, exhibits high expression levels on naive T/B cells and DCs, and it can coordinate inflammatory responses while regulating the migration and function of white blood cells (Salem et al., 2021). Our results suggested that the infiltration proportion of naive T/B cells was reduced in AML and confirmed low expression of CCR7 in AML clinical samples.

In addition, CCR7 has been confirmed to be related to multiple tumors (Zlotnik et al., 2011; Takanami, 2003; Sperveslage et al., 2012; Shang et al., 2009). DCs are vital to maintain immune homeostasis. Studies demonstrated that the dysfunction of DCs can damage the immune response of AML (Rickmann et al., 2013; Lau et al., 2016). As effective antigen-presenting cells, DCs induce antigens and migrate to the draining lymph nodes, thereby activating the immune response (Tiberio et al., 2018). In this study, we proved that CCR7 was markedly positively associated with the activation of DCs, consistent with the findings by Ohl et al. (2004). Therefore, we supposed that in AML patients, downregulating CCR7 may weaken the antigen-presenting ability of DCs, thereby damaging the inflammatory response and reshaping the immune microenvironment.

MS4A1 encodes a 33–37-kDa non-glycosylated protein CD20, which is present on both normal and malignant B lymphocytes (Tedder et al., 1989). The expression of CD20 exhibits high heterogeneity in different tumors. More than 20% of B-cell precursor lymphoma patients exhibit high expression of CD20 (Jeha et al., 2006). On the contrary, MS4A1 is downregulated in some tumors, like breast cancer and colorectal cancer (Milne et al., 2009; Mudd et al., 2021; Han et al., 2008). Additionally, we proved that MS4A1 was highly correlated with the immune infiltration of tumor, so we assumed that MS4A1 may improve patient prognosis by regulating immune homeostasis. Sato et al. proved that patients with high expression of CD20 tumor-infiltrating cells have good prognosis in thymic cancer (Sato et al., 2020). In addition, CD8^+^ T cells serve as the primary effector cells in anti-tumor immunity, and their substantial infiltration into the TIME inhibits the progression and growth of cancer. Song et al. found that MS4A1 was highly expressed in CD8^+^ T cells (Song et al., 2022), which further indicated that MS4A1 was related to the tumor immune microenvironment.

SLC16A6 belongs to the solute carrier family. Current research has not yet characterized the relationship between SLC16A6 and the immune system, but evidence suggests that SLC16A6 participates in taurine transport and may promote the release of taurine from cytoplasmic membranes. Taurine functions as a key organic osmolyte, playing a dual role in both regulating cell volume and modulating immune responses (Higuchi et al., 2022). It was proved that the lack of taurine in CD8^+^ T cells leads to cell death and dysfunction, inducing an immunosuppressive microenvironment. In other words, supplementation with taurine can effectively restore T-cell function, inducing anti-tumor immune responses (Cao et al., 2024). Therefore, we reasonably assumed that immune-infiltrating cells with SLC16A6 were downregulated in AML patients, leading to taurine depletion and reduced immune response.

In addition, our research found that three genes were significantly under-expressed in LSC^+^ cells and in relapsed patients, which has clinical implications for prognosis and has not been reported in previous studies. Finally, using clinical samples, compared with the healthy controls, the expressions of three genes were markedly reduced in AML patients. Nevertheless, our study still has limitations. Even though we have demonstrated that, in AML patients, hub genes may regulate the immune microenvironment, the underlying mechanism still needs to be explored experimentally. In conclusion, our study identified and validated CCR7, SLC16A6, and MS4A1 as tumor suppressors involved in the development of AML and related to immune cell infiltration.

## 5 Conclusion

In this study, we identified and validated three tumor suppressors (SLC16A6, CCR7, and MS4A1) in AML; they were markedly reduced in AML patients. It was demonstrated that three genes may influence the TIME by modulating immune cell infiltration and contributing to the regulation of cytokines. In addition, patients with high expression of three genes consistently displayed better prognosis, whereas those with low expression typically faced shorter survival times. These findings will provide novel biomarkers for AML and offer new insights into precision therapy.

## Data Availability

The datasets presented in this study can be found in online repositories. The names of the repository/repositories and accession number(s) can be found in the article/Supplementary Material.

## Supplementary material

SUPPLEMENTARY FIGURE 1
**(A–C)** Correlation analysis between the expression levels of three hub genes and the molecular characteristics of AML patients in GSE37642, including Runx1–Runx1t1 fusion, Runx1 mutation, and FAB subtype.
