# Supplementary material for: Identification and validation of three tumor suppressors associated with the immune response of acute myeloid leukemia
Source: Front Genet. 2025 Sep 16;16:1652142. doi: 10.3389/fgene.2025.1652142 (PMC12479338; doi:10.3389/fgene.2025.1652142)
Supplement: Supplementary file 1 [file Table1.docx]

| Patient ID | Gender | Age | Detected molecular features |
| --- | --- | --- | --- |
| 1 | male | 42 | M5, ASXL1 mutation |
| 2 | male | 64 | M0, DNMT3A, IDH1, NPM1, PTPN11 mutation, WT1+ |
| 3 | female | 78 | M2, 46, XX, -7, add(11) (p15), -12, -16, -17, -17, -18, +1-4mar{cp16} |
| 4 | male | 69 | M0, Normal karyotype, FLT3 mutation 12.51%, SF3B1 mutation 33.68%, ASXL1 mutation 12.31%, Runx1 mutation 39.56% |
| 5 | female | 59 | M5, DNMT3A+, PTPN11+, WT1+ |
| 6 | female | 34 | M5, (NPM1, NRAS, PTPN11, DNMT3A, IDH1, KMT2D, SF1) mutation, WT1+ |
| 7 | male | 45 | M0, Genetic testing was not performed. |
| 8 | male | 63 | M4, WT1+, IDH2+, NPM1+ |
| 9 | male | 21 | M2,46, XY, Normal karyotype, (DNMT3A, FLT3, RUNX1, SF3B1) mutation |
| 10 | female | 58 | M5, +8, +8, add(15), (p11), +22, del (22), (q13),{7} |
| 11 | male | 67 | M2, WT1+, AML1-ETO mutation |
| 12 | female | 29 | M0, IDH1+, NPM1+, PTPN11+ |
| 13 | male | 82 | M5, (FLT3, ITD, NPM1, TET2, ZRSR2) mutation |

Supplementary Table1 Clinical and molecular characteristics of 13 AML patients.
